# Staphylococcal resistance profiles in deep infection following primary hip and knee arthroplasty: a study using the NJR dataset

**DOI:** 10.1007/s00402-019-03155-1

**Published:** 2019-03-15

**Authors:** Richard J. Holleyman, David J. Deehan, Lucy Walker, Andre Charlett, Julie Samuel, Mark D. F. Shirley, Paul N. Baker

**Affiliations:** 1 Institute of Cellular Medicine, Newcastle University, Newcastle upon Tyne, NE2 4HH UK; 2 Health Education North East, Newcastle upon Tyne, NE15 8NY UK; 3 Newcastle upon Tyne NHS Foundation Trust, Newcastle upon Tyne, NE7 7DN UK; 4 Centre for Infectious Disease Surveillance and Control, Public Health England, London, NW9 5EQ UK; 5 Biological, Clinical, and Environmental Systems Modelling Group, School of Biology, Newcastle University, Newcastle upon Tyne, NE1 7RU UK; 6 Department of Trauma and Orthopaedics, James Cook University Hospital, Middlebrough, TS4 3BW UK

**Keywords:** Arthroplasty, Revision, Infection, Sensitivity, Resistance, Staphylococcus

## Abstract

**Introduction:**

This study aimed to (1) report the rates of resistance against a variety of antibiotics for pure Staphylococcal infections, and (2) examine the impact of ALBC use at primary surgery has on resistance patterns for patients undergoing first-time revision of primary hip and knee arthroplasty for indication of infection.

**Materials and methods:**

Data from the National Joint Registry database for England and Wales were linked to microbiology data held by Public Health England to identify a consecutive series of 258 primary hip and knee arthroplasties performed between April 2003 and January 2014 that went on to have a revision for Staphylococcal deep periprosthetic infection. Multivariate binary logistic regression was used to study predictors of microorganism resistance to a range of antimicrobials.

**Results:**

After adjusting for patient and surgical factors, multivariate analysis showed the use of gentamicin-loaded bone cement at the primary surgery was associated with a significant increase in the risk of Staphylococcal gentamicin resistance (odds ratio 8.341, 95% CI 2.297–30.292, *p* = 0.001) and methicillin resistance (odds ratio 3.870, 95% CI 1.319–11.359, *p* = 0.014) at revision for infection.

**Conclusions:**

Clinicians must anticipate the possibility of antibiotic resistance to ALBC utilised at primary surgery.

## Introduction

Deep periprosthetic infection (PJI) following primary hip and knee arthroplasty is a catastrophic complication in terms of patient outcome and cost to the health service. The implicated microorganism(s) are important determinants of clinical outcome and previous work by our group has described the epidemiology in England and Wales by linking data held by the national joint registry (NJR) to national microbiology data [11, 12].

In addition to identifying the microorganisms implicated in deep periprosthetic infection, it is vital to establish their antimicrobial resistance and susceptibility patterns to guide both prophylactic and therapeutic antibiotic therapies. Such therapies include systemic antibiotics (in oral and intravenous form) and local antibiotic delivery via antibiotic-loaded bone cement (ALBC) that allows delivery of high concentrations of antibiotics to the surgical site while reducing systemic side effects. However, while the use of ALBC is widespread, there is evidence to suggest that its use may lead to resistant bacterial strains [3, 15, 18].

Using data from the National Joint Registry for England and Wales and Public Health England, this study therefore aimed to: (1) report the rates of resistance against a variety of antibiotics for pure Staphylococcal infections, and (2) examine the impact of ALBC use on resistance patterns for patients undergoing first-time revision of primary hip and knee replacement for indication of infection.

## Patients and methods

**Fig. 1:**
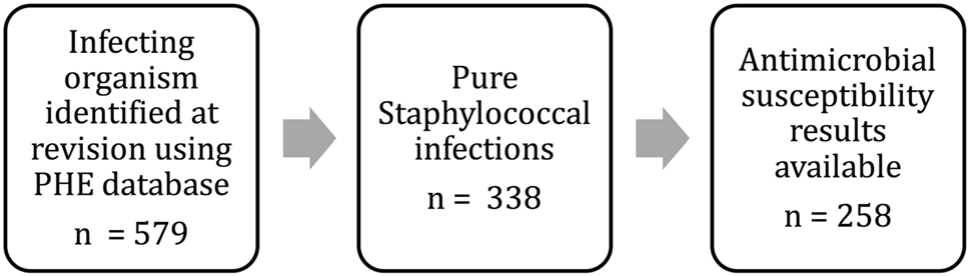
Description of the study cohort

This retrospective study linked two national databases to study antimicrobial susceptibility and resistance patterns in first-time revision for Staphylococcal deep periprosthetic infection. The methodology used to identify cases revised for periprosthetic joint infection (PJI) from the National Joint Registry for England, Wales and Northern Ireland (NJR) and subsequent linkages to microbiological data from Public Health England (PHE) has been described previously [11–13]. From the linked cohort of 579 cases (248 hips, 331 knees) a total of 258 (45%) infected arthroplasties (105 hips, 153 knees) were identified with a pure Staphylococcal periprosthetic infection for whom antimicrobial susceptibilities were available (see Fig. 1). 157 of the cohort were male (61%) with a mean age of 68.3 years (range 18–98 years). The majority of patients were ASA grade 2 or below (ASA 1, *n* = 31, 12%; ASA 2, *n* = 176, 68%).

### Dataset preparation

Diagnosis of periprosthetic infection was determined based upon the ‘reason for revision’ indication recorded by the surgeon contemporaneously at the time of surgery and subsequently uploaded to the NJR. The recording of a case as infected is therefore based upon preoperative clinical review, relevant biochemical testing and preoperative microbiological investigation, as well as any perioperative surgical findings.

### Antimicrobial susceptibility analysis

The study cohort of 258 cases contained more than 3,000 antimicrobial susceptibility tests. Staphylococcal susceptibility tests against each antimicrobial were classified as ‘sensitive’, ‘intermediate’, or ‘resistant.’ ‘Intermediate’ results comprise < 0.5% of results and were therefore excluded. For some patients, there were multiple tests against the same antimicrobial (e.g.,0020due to multiple samples sent at revision surgery); in such cases, where any test result was ‘resistant’ that patient’s infection was deemed resistant to the given antimicrobial (even if other samples were reported as ‘sensitive’).

Using the NJR data, cases were classified according to the use of bone cement. We defined any use of bone cement at primary surgery (including hybrid combinations) as cemented fixation. Cement types were further sub-classified according to their antibiotic content.

### Statistical analysis

The null hypothesis was that, for a range of antimicrobials, the use of gentamicin-loaded bone cement at primary surgery would not affect Staphylococcal antimicrobial susceptibility pattern observed at time of revision. In total 31 individual antibiotics were tested against the identified samples. For the purpose of analysis we wanted to identify a group of antimicrobials for which we could analyse the influence of ABLC upon subsequent organism resistance. This was done based on the following criteria (a) antimicrobials with a minimum number of 100 antimicrobial susceptibility tests and (b) tests in which the observed proportion of resistant cases was > 10%. These values were chosen to allow meaningful numbers and group sizes within our regression analysis. Preliminary analysis demonstrated that the cohort of knee arthroplasties was cemented in more than 95% of cases. The comparison group (uncemented) was therefore too small to allow robust regression analysis. As such, only hip arthroplasties were included in the regression analysis.

For each antimicrobial, a binary logistic regression model was created with resistance as the outcome and predictors being (1) the use of gentamicin-loaded bone cement vs. uncemented fixation; (2) age (grouped by < 55, 55–65, 65–75, > 75 years); (2) ASA grade (ASA 1, ASA 2, ASA 3 or 4); (3) gender; (4) body mass index (grouped by < 25, 25–30, 30–40, > 40 kg/m2) and (5) indication for primary surgery. Both univariate and multivariate models were performed. A *p* value of < 0.05 was deemed to be statistically significant.

### Ethical approval and data governance

Data for this study were accessed using the standard NJR research process. The research request for this study (I.D. no. D2072003) was approved by the NJR research committee on 1 May 2014. Data linkage was achieved directly between the NJR and Public Health England. As such, no patient sensitive information (NHS no.) was held by Newcastle University as part of this analysis. After methodological review by Public Health England’s confidentiality advisory group, it was determined that ethical approval was not required and the project was therefore undertaken as a service evaluation. The authors declare that they have no conflict of interest with respect to this study which received no funding.

## Results

Susceptibility profiles for each antimicrobial tested are shown in Table 1 for the cohort of 258 arthroplasties. Not all antimicrobials were tested for in all cases. Resistance rates varied from 0% to more than 96%.

**Table 1:** Antimicrobial resistance patterns for cohort of hip and knee arthroplasties revised for Staphylococcal infection (*n* = 258)

Antibiotic	No. of cases	No. resistant	% resistant
Amikacin	13	2	15.4
Amoxycillin/clavulanate	25	10	40.0
Ampicillin_amoxycillin	27	26	96.3
Ceftriaxone	5	4	80.0
Cephalexin	11	4	36.4
Chloramphenicol	103	0	0.0
*Ciprofloxacin	231	71	30.7
Clarithromycin	56	21	37.5
*Clindamycin	163	36	22.1
Co-trimoxazole	39	7	17.9
Daptomycin	51	3	5.9
*Erythromycin	227	80	35.2
Fosfomycin	21	3	14.3
*Fusidic acid	249	83	33.3
*Gentamicin	251	74	29.5
Imipenem	5	4	80.0
Levofloxacin	16	3	18.8
Linezolid	208	1	0.5
*Methicillin	253	100	39.5
Minocycline	1	0	0.0
Moxifloxacin	4	0	0.0
Mupirocin	206	20	9.7
Nitrofurantoin	29	1	3.4
*Penicillin	182	155	85.2
Rifampicin	239	23	9.6
Teicoplanin	173	16	9.2
*Tetracycline	246	44	17.9
Tigecycline	66	0	0.0
Tobramycin	31	12	38.7
*Trimethoprim	180	63	35.0
Vancomycin	208	3	1.4

Asterix indicates antibiotics tested in regression analysis (> 10% resistance, > 100 cases)

### Impact of gentamicin-loaded bone cement

Staphylococcal resistance patterns for antimicrobials fulfilling the inclusion criteria (ciprofloxacin, clindamycin, erythromycin, fusidic acid, gentamicin, methicillin, penicillin, tetracycline and trimethoprim) were compared according to the use of either gentamicin-loaded bone cement or uncemented fixation at primary hip arthroplasty. For the majority of antimicrobials tested, an increased rate of resistance was observed in association with the use of gentamicin-loaded bone cement. This was most evident for gentamicin where the use of gentamicin-loaded bone cement was associated with gentamicin resistance in 59 of 166 cases (36%) as compared to 6 of 49 cases (12%) where purely uncemented fixation was employed at the primary procedure (Tables 2, 3).

**Table 2:** Antimicrobial susceptibility profile for hip arthroplasties using an uncemented vs. gentamicin-loaded bone cement fixation at primary surgery

Antibiotic	Gentamicin cement						Uncemented					
	Number of cases	No. resistant	% resistance	Mean age	% male	Median time to revision (years)	Number of cases	No. resistant	% resistance	Mean age	% Male	Median time to revision (years)
Ciprofloxacin	39	16	41.0	71.7	67	2.6	40	9	22.5	61.5	65	2.3
Clindamycin	28	4	14.3	72.7	61	2.6	30	2	6.7	63.6	63	2.3
Erythromycin	41	18	43.9	70.6	66	1.6	41	9	22.0	61.8	63	2.5
Fusidic_acid	42	12	28.6	71.1	67	2.2	44	13	29.5	62.0	64	2.3
Gentamicin	44	20	45.5	71.7	66	2.2	44	6	13.6	62.0	64	2.3
Methicillin	45	22	48.9	71.1	67	1.6	44	10	22.7	62.0	64	2.3
Penicillin	38	33	86.8	71.7	61	1.7	30	26	86.7	61.7	67	2.5
Tetracycline	43	8	18.6	71.3	63	1.6	44	5	11.4	62.0	64	2.3
Trimethoprim	34	11	32.4	72.8	65	2.6	34	7	20.6	62.4	65	2.6
Total	354	144	40.7	71.6	65	1.8	351	87	24.8	62.0	64	2.5

**Table 3:** Antimicrobial susceptibility profile for knee arthroplasties using an uncemented vs. gentamicin-loaded bone cement fixation at primary surgery

Antibiotic	Gentamicin cement						Uncemented					
	Number of cases	No. resistant	% resistance (%)	Mean age	% Male	Median time to revision (years)	Number of cases	No. resistant	% resistance	Mean age	% Male	Median time to revision (years)
Ciprofloxacin	116	34	29.3	69.8	56	1.6	4	1	25.0	66.3	75	2.6
Clindamycin	82	25	30.5	68.7	48	1.5	1	0	0.0	53.1	100	2.1
Erythromycin	110	43	39.1	69.4	55	1.8	5	1	20.0	68.7	60	2.1
Fusidic_acid	122	44	36.1	69.7	57	1.6	5	0	0.0	68.7	60	2.1
Gentamicin	122	39	32.0	69.4	57	1.6	5	0	0.0	68.7	60	2.1
Methicillin	124	56	45.2	69.7	58	1.6	5	0	0.0	68.7	60	2.1
Penicillin	85	73	85.9	70.3	60	1.5	4	2	50.0	71.2	50	2.6
Tetracycline	120	25	20.8	69.8	58	1.6	4	1	25.0	66.3	75	2.6
Trimethoprim	85	34	40.0	70.2	56	1.5	3	1	33.3	64.4	100	2.1
Total	966	373	38.6	69.7	56	1.5	36	6	16.7	67.6	67	2.1

For the cohort of infected hip arthroplasties, univariate binary logistic regression analysis demonstrated that the use of gentamicin-loaded bone cement at primary surgery was associated with significantly increased risk of resistance at revision surgery to erythromycin (odds ratio 2.73, 95% CI 1.062–7.289, *p* = 0.037), gentamicin (odds ratio 5.278, 95% CI 1.855–15.017, *p* = 0.002) and methicillin (odds ratio 3.252, 95% CI 1.301–8.127, *p* = 0.012). After adjusting for patient and surgical factors, multivariate analysis showed the use of gentamicin-loaded bone cement was associated with an eightfold increase in the risk of gentamicin resistance (odds ratio 8.341, 95% CI 2.297–30.292, *p* = 0.001) and almost four times the risk of methicillin resistance at revision for infection (odds ratio 3.870, 95% CI 1.319–11.359, *p* = 0.014). The use of antibiotic-loaded bone cement had no significant effect on the rates of Staphylococcal resistance to penicillin, fusidic acid, tetracycline, ciprofloxacin, erythromycin, trimethoprim or clindamycin at revision surgery when compared to uncemented fixation (Table 4).

**Table 4:** Binary logistic regression analysis showing odds ratio for resistance to the named antibiotic given the use of gentamicin-loaded bone cement compared to uncemented fixation at primary HIP surgery

Antibiotic	Univariate			Multivariate		
	Odds ratio	95% CI	*p* value	Odds ratio	95% CI	*p* value
Ciprofloxacin	2.396	0.900–6.376	0.08	1.901	0.625–5.779	0.258
Clindamycin	2.333	0.392–13.875	0.352	0.573	0.022–14.767	0.737
Erythromycin	2.783	1.062–7.289	0.037*	2.329	0.795–6.826	0.123
Fusidic acid	0.954	0.376–2.421	0.921	0.974	0.333–2.847	0.961
Gentamicin	5.278	1.855–15.017	0.002**	8.341	2.297–30.292	0.001**
Methicillin	3.252	1.301–8.127	0.012*	3.87	1.319–11.359	0.014*
Penicillin	1.015	0.247–4.166	0.983	0.715	0.115–4.467	0.72
Tetracycline	1.783	0.533–5.961	0.348	2.476	0.624–9.832	0.198
Trimethoprim	1.845	0.615–5.535	0.275	2.682	0.729–9.872	0.138

*p < 0.05, **p < 0.01

## Discussion

The principal finding of this study was that for patients undergoing revision of a primary hip arthroplasty due to Staphylococcal deep infection, there was a significantly increased rate of resistance to gentamicin and methicillin in association with use of gentamicin-loaded bone cement at primary surgery. This study did not identify any further significant patient or surgical predictors of Staphylococcal antibiotic resistance. Clinicians should be suspicious of gentamicin resistance in all cases where antibiotic-loaded bone cement is used at primary surgery and furthermore, in such cases, use of gentamicin-loaded cement may not be appropriate as a single local antibiotic therapy at revision surgery.

There is strong registry and randomised controlled trial evidence for the efficacy of ALBC in reducing the rate of both septic and aseptic revision hip and knee arthroplasty [5, 8]. A meta-analysis by Parvizi et al. [20] reported that the use of antibiotic-impregnated cement lowered periprosthetic infection rate by approximately 50% in primary hip arthroplasty and for revisions of previously infected hips, combinations or culture-dependent antibiotics lowered infection rates by approximately 40%. Although there is an expanding body of work that demonstrates an increased rate of antibiotic resistance following the use of antibiotic-loaded bone cement, this is at odds to other studies which refute a significant relationship [7]. Hansen et al. [10] found no significant change in the epidemiology of infecting pathogens nor any notable increase in percentage resistance among organisms isolated from patients with deep infection that had received prophylactic antibiotic-loaded cement in their primary joint arthroplasty. Whether the benefit of antibiotic-loaded cement in managing and preventing deep periprosthetic infection outweighs any negative effects from subsequent resistance is unknown. However, as microorganism prevalence and resistance patterns evolve there may be a need for a greater emphasis on the choice of antibiotic used within ALBC.

Previous animal research has demonstrated an increase in gentamicin-resistant infections in rats impregnated with gentamicin-loaded cement pellets [25].

Antimicrobial resistance can occur in a variety of ways [17]. Mutational resistance can occur with various antibiotic-organism combinations. Translocation of resistance genes to mobile DNA is rarer, but can cause ‘strain epidemics’. Furthermore, existing clones of resistant bacteria may simply be selected out in an antibiotic-rich environment. The process by which an antibiotic is rendered ineffective against a particular bacterial strain may also be related to the use of orthopaedic biomaterials as well as the ALBC [1, 2, 9]. ALBC has an optimum surface for colonisation, and prolonged exposure to antibiotics at sub-inhibitory levels allows mutational resistance to occur [15, 16, 19, 22, 25]. In vivo cement use (where antibiotic-loaded bone cement will remain in situ for many years), is associated with continued elution of sub-therapeutic levels of antibiotic for many years and this may further increase microbial resistance rates in these cases [4, 21, 23].

Corona et al. examined the impact of aminoglycoside-cement spacers on aminoglycoside resistance in a cohort of 113 chronic periprosthetic infections following hip and knee arthroplasty due to Gram-positive cocci [6]. They reported overall gentamicin and tobramycin resistance to be 32 and 41%, respectively. Additionally they found gentamicin and tobramycin resistance at re-revision to be significantly higher following the use of aminoglycoside loaded cement spacers.

In the univariate analysis, erythromycin resistance was significantly higher in patients in the ALBC group. However, after controlling for other covariates (age, gender, ASA grade, BMI, and primary surgery) the effect of ALBC as a predictor of erythromycin resistance was no longer significant. This can be attributed to the small predictive effect of ALBC of erythromycin as evidenced by the 95% confidence intervals in the univariate analysis (Table 4) becoming overwhelmed by the effects of other covariates. The methodological and data linkage limitations associated with the present study have been discussed previously [11, 12]. Unfortunately, the small numbers of cases employing uncemented fixation in the knee arthroplasty cohort precluded meaningful regression analysis of predictors of antibiotic resistance in this group. Antimicrobial susceptibility data were missing for 80 of 338 (24%) of cases of Staphylococcal PJI. Antimicrobial susceptibility data submission is not mandatory for individual laboratories and whilst we cannot rule out selection bias, we believe the risk of this to be low as data submission to PHE is not conditional on the particular antimicrobial susceptibility profile for a given sample.

Notwithstanding the above limitations, the findings of our study are supported by existing clinical studies. Weber and Lautenbach identified gentamicin resistance in 29% of bacteria isolated preoperatively in patients revised for periprosthetic infection; this rose to 40% in cases where gentamicin-loaded bone cement was used. In a cohort of 91 cases, Hope et al. [14] found bacterial strains resistant to gentamicin in 88% of cases of prosthetic infection in primary hip arthroplasty where cement was loaded with this antibiotic as compared to 16% found after those where other cement types were used. An increased re-infection rate was also seen in patients presenting with a gentamicin-resistant infection. Whilst they did not study the impact of antibiotic bone cement, Stefansdottir et al. [24] performed a comprehensive analysis of 426 knee arthroplasties revised for infection using data from the Swedish arthroplasty register. Although antimicrobial susceptibility data were incomplete, the study identified a gentamicin resistance rate of approximately 34% (20 of 58 cases of Staphylococcal infection) which compares favourably to an incidence of 29.5% (74 of 251 cases) observed in our study. They also reported an increase in the rate of methicillin resistance over time.

In this study we were unable to report microorganism at the species level, thus there may be cases of methicillin-resistant *Staphylococcus aureus* (MRSA) PJI responsible for several of the observed cases of methicillin resistance. Furthermore, a significant proportion of Staphylococcal infections will be represented by Coagulase-negative staphylococci (CoNS) of which a proportion will be methicillin resistant and hence cross resistance with gentamicin and erythromycin is not unexpected; it is possible that gentamicin ALBC may lead to a selection bias for CoNS and hence the observed increase in the proportion of resistant cases may be due to an organisms’ selection bias rather than development of de novo resistance within infecting organism.

In conclusion, our study reported the resistance patterns of Staphylococci strains to a broad range of antibiotics and examined the impact of gentamicin-loaded bone cement on the resultant antimicrobial susceptibility profile. Multivariate analysis showed the use of gentamicin-loaded bone cement was associated with an eightfold increase in the risk of gentamicin resistance and almost four times the risk of methicillin resistance at revision for infection. Clinicians must therefore remain vigilant to the possibility of antibiotic resistance to a given ALBC utilised at primary surgery when deciding both local and systemic antibiotic choice in the treatment of PJI at time of presentation and during revision surgery.
